# Editorial: Agricultural planting improvement and risk control

**DOI:** 10.3389/fbioe.2025.1577861

**Published:** 2025-03-11

**Authors:** Naeem Khan, Weicong Qi, Yi Han, Xiaofeng Su, Kathleen L. Hefferon

**Affiliations:** ^1^ Agronomy Department, University of Florida, Gainesville, FL, United States; ^2^ Provincial Key Laboratory of Agrobiology and Institute of Germplasm Resources and Biotechnology, Jiangsu Academy of Agricultural Sciences, Nanjing, China; ^3^ School of Life Sciences, Anhui Agricultural University, Hefei, China; ^4^ Institute of Biotechnology, Chinese Academy of Agricultural Sciences, Beijing, China; ^5^ Department of Microbiology, Cornell University, Ithaca, NY, United States

**Keywords:** agriculture, plant and soil, sequencing, genome editing, data minig

Agriculture remains at the core of human society, influencing food security, global economies, and environmental sustainability. Despite continuous advancements in agricultural technologies, global challenges such as widespread starvation, malnutrition, and the looming threats of climate change persist, highlighting the urgent need for more efficient and environmentally sustainable farming practices. Traditional agricultural systems still rely heavily on herbicides, pesticides, and chemical fertilizers, all of which contribute to environmental degradation. As the world faces unprecedented environmental shifts, the call for innovative agricultural solutions that minimize the use of harmful chemicals and maximize land resource efficiency has never been more pressing.

The dawn of the 21st century has seen an explosion of groundbreaking technologies in fields like genomics, biotechnology, and artificial intelligence. These innovations hold great promise for transforming traditional agricultural strategies, enabling the development of new approaches to address the pressing challenges facing global agriculture. Technologies such as high-throughput nucleotide sequencing, genome editing, and artificial intelligence are poised to revolutionize crop cultivation, gardening, and soil remediation by optimizing planting patterns, improving resource use efficiency, and adapting to changing environmental conditions.

One area that has garnered significant attention is the role of plant hormones in improving agricultural productivity. For example, a study by Sekhukhune and Maila explored the effects of exogenous indole-3-butyric acid (IBA) on root formation in kiwifruit stem cuttings. This research demonstrated that varying concentrations of IBA had significant impacts on root development in two kiwifruit species (*Actinidia deliciosa* and *Actinidia arguta*). Specifically, 10,000 ppm of IBA resulted in the highest rooting percentage and root length in *A. deliciosa*, while 100,000 ppm yielded the greatest number of roots. This study emphasizes the potential of plant growth regulators to enhance propagation techniques and improve the overall growth outcomes of crops.

In addition to plant hormone research, the exploration of plants’ genetic and epigenetic responses to environmental stressors is another important area of agricultural science. For instance, Wang et al. investigated the epigenetic response of foxtail millet (*Setaria italica*) to elevated ozone (O3) stress. Their study demonstrated that exposure to ozone caused significant physiological damage, including leaf yellowing, curling, and necrotic spots. Through Methylation Sensitive Amplified Polymorphism (MSAP) analysis, they revealed that ozone exposure led to DNA methylation changes in both the S0 and S1 generations of millet. The S1 generation showed a marked reduction in overall genome methylation, particularly at CNG sites, and displayed stable inheritance of methylation patterns for specific genes. These findings offer new insights into how plants adapt to environmental stress and the role of epigenetic modifications in facilitating such adaptation.

Another area where innovation has been applied is in optimizing crop growth through the use of strategic planting patterns. Li et al. focused on optimizing the growth of daylilies (*Hemerocallis citrina Baroni*) by modeling the effects of various planting configurations on bud yield and land resource utilization. Their study utilized partial least squares path modeling (PLS-PM) to analyze how different planting configurations impacted light interception, photosynthesis, and resource efficiency. After simulating 36 planting scenarios, the researchers identified an optimal planting configuration, with a plant density of 83,000 plants·ha^-1^ and row spacing of 0.8 m. The results suggested that although planting patterns had a relatively minor effect on yield, using a combination of wide and narrow row spacing could help improve yield. These findings contribute valuable knowledge to daylily production and land use optimization strategies.

In the field of disease control, Ren et al. developed a novel rice seed-coating agent combining phenamacril and fludioxonil to manage rice diseases, particularly bakanae and seedling rot, in the Yangtze River Basin. The antifungal activity of six different fungicides was assessed, with phenamacril and fludioxonil proving highly effective against *Fusarium moniliforme* and *Curvularia lunata*. The study also investigated the impact of these fungicides on rice seed germination and seedling growth. The new seed-coating agent demonstrated minimal toxicity to zebrafish, superior disease control with a 84.83% efficacy rate against bakanae, and up to a 30.48% increase in rice yield compared to traditional seed treatments. This breakthrough offers a safer, more sustainable alternative to conventional chemical treatments and presents significant cost-saving potential for rice farmers.

Finally, the role of microorganisms in soil health and plant growth has also been explored. Wei et al. conducted a study on the rhizospheric and non-rhizospheric microorganisms of *Poa alpigena L.* in the Qinghai Lake basin. The research identified a diverse microbial community, with 5,681 species across 1,606 genera. The study found that non-rhizospheric microorganisms had higher species richness, while the abundance of dominant microorganisms was similar in both rhizospheric and non-rhizospheric soils. The phylum *Proteobacteria* dominated both soil types, with significant differences in the microbial composition between the two soil environments. Rhizospheric microorganisms were enriched in pathways related to antibiotic biosynthesis and methane metabolism, suggesting that these microorganisms could play a role in mitigating global warming by controlling methane emissions. This study highlights the importance of understanding soil microbiomes to enhance agricultural productivity and reduce environmental impact.

In conclusion, these studies illustrate the immense potential of integrating innovative technologies, sustainable practices, and scientific research to enhance agricultural productivity and sustainability. By improving planting strategies, controlling diseases more effectively, and leveraging plant genetic and microbial resources, the agricultural sector can meet the growing global demand for food while addressing environmental and climate challenges. This holistic approach to agricultural development is key to securing a more sustainable and resilient future for global food production.

